# Effect of the Application of a Dehydrothermal Treatment on the Structure and the Mechanical Properties of Collagen Film

**DOI:** 10.3390/ma13020377

**Published:** 2020-01-14

**Authors:** Xuefei Chen, Lingling Zhou, Huaizhong Xu, Masaki Yamamoto, Masaya Shinoda, Masanori Kishimoto, Tomonari Tanaka, Hideki Yamane

**Affiliations:** 1 Department of Biobased Materials Science, Kyoto Institute of Technology, Kyoto 606-8585, Japan; cxf2140089@163.com (X.C.);; 2 Textile Research Institute of Gunma, Gunma 376-0011, Japan; 3 Nitta Gelatin Inc., Osaka 581-0024, Japan

**Keywords:** collagen film, dehydrothermal treatment, crosslinking, denaturation, mechanical property, structure

## Abstract

Dehydrothermal (DHT) treatment was used to improve the properties of collagen casings because of its non-cytotoxicity. Understanding the effects of DHT treatment on the structure and mechanical properties of collagen films is beneficial to developing satisfying collagen casings. Herein, DHT treatment with various temperatures (85–145 °C) and timescales (1–7 days) were investigated. It was clarified that the chemical crosslinking covalent bond between collagen molecules was formed after the DHT treatment. Crosslinking density increased with increasing DHT treatment temperatures, contributing to the increase of tensile strength up to over three times of that of the untreated collagen film. The increased crosslinking density was also found when increasing the DHT treatment time, and the maximum was obtained in 3 days. Further DHT treatment time did not change the crosslinking density. The damage in the triple helix structure and the self-assembly of collagen molecules were observed from IR and SAXS. The extent of denaturation increased with increasing DHT treatment temperature and time, although the effect of the DHT treatment time on the denaturation was more moderate. When the DHT treatment temperature was as high as 145 °C or the DHT treatment time exceeded 5 days, serious denaturation occurs, leading to the deterioration of mechanical properties.

## 1. Introduction

Biocompatible, biodegradable, and edible biomaterials like protein have received a great deal of attention in wound dressing, scaffolds, and the food industry recently [1,2]. Collagen, among the potential biomaterials, is an abundant fibrous protein, which accounts for 30% of all vertebrate bodies and provides structural integrity in connective tissues [3]. There are at least 29 types in the collagen super family, of which fibrillar structure collagen represents over 90% [4]. In type I collagen, two identical alpha chains, called the α1−chain and the third chain called α2−chain, are twisted with each other into a right-handed triple helix structure. Every chain contains the repeating amino acids (Gly-X-Y), where the X and Y are frequently proline and hydroxyproline [5]. Collagen molecules organize themselves to fibrils side by side with intrafibrillar crosslinking. The well-organized self-assembly aggregation of collagen molecules gives a striation on the surface of fibril, the periodicity of which striation is about 64 nm based on different origins [6]. These fibrils can form into fibers which can interweave into one-dimensional networks or two- and three-dimensional networks according to different functions [7].

Collagen casings are tubular collagen films widely used as edible artificial sausage casings. As such, the artificial collagen casings hold almost half of the market share in the sausage industries because of their uniformed size, sanitary nature, etc. [8]. However, our previous study showed that the artificial film prepared from natural collagen has poor mechanical properties and a high swelling ratio because of the layered structure with a large gap filled with fine collagen fibrils [9]. Cellulose is usually mixed with collagen as a reinforcement to enhance the mechanical properties of collagen materials, since it forms electrostatic and hydrogen bonding interactions with collagen to have a dense network structure [10,11,12]. On the other hand, the swelling ratio of collagen materials in water also increases with the addition of cellulose due to the hydrophilicity of cellulose [11].

Crosslinking is an important approach to improve the mechanical properties of collagen materials and to make the degree of swelling lower. Dehydrothermal (DHT) treatment, one of the physical crosslinking methods, is generally used to obtain excellent properties of collagen materials without introducing extra reagents [13]. The process of DHT treatment is mainly used to expose the collagen to a high temperature for a period in a vacuum and to remove water to form the crosslinks. Yannas et al. [14] and Silver et al. [15] showed that the number of free acidic and basic residues on collagen molecules decreased with DHT treatment and suggested that the crosslinks is the result of a condensation reaction. Furthermore, the removal of water from collagen may damage original hydrogen bonds in collagen, resulting in the introduction of an amide–amide hydrogen bond, which is stronger than amide–water hydrogen bond, and improves the properties of the collagen materials [16]. Understanding the precise mechanism of crosslinking is necessary for the appropriate application of DHT treatment in the collagen materials.

The temperature and the period of DHT treatment are key factors in improving the properties of collagen materials. Gorham et al. reported that a higher temperature and a longer period are beneficial to increasing the crosslinking density [17]. In addition, denaturation of collagen is also sensitive to these factors. Denaturation damages the triple helix configuration and alters the collagen molecules packing, which may deteriorate the mechanical properties of collagen [6]. It is meaningful to clarify the relationship between the formation of crosslinking and the occurrence of the denaturation. This is critical to obtain the favorable mechanical properties of collagen materials. Haugh et al. used infrared spectroscopy to analyze the relationship among the tensile modulus, the degree of crosslinking, and the extent of the denaturation [18]. Yunoki et al. evaluated the extent of denaturation upon the application of DHT treatment at several specific temperatures for various periods of time without showing any information regarding mechanical properties [19]. Our previous work had shown that DHT treatment can improve the tensile strength of collagen film by more than three times under selected conditions, whereas the occurrence of denaturation was detected simultaneously [9]. To the best of our knowledge, there is no more information available about the effect of the relation between the extent of denaturation and the degree of crosslinking on the mechanical properties of collagen films in various DHT temperatures and timescales.

The effect of DHT treatment, with various conditions on the structure and properties of collagen films, was investigated in this study to develop satisfying collagen casings. The nature of crosslinking and the variation of crosslinking density were analyzed by evaluating the swelling ratios of collagen films in deionized water and in aq. urea. The denaturation of collagen after various DHT treatments was analyzed systematically by using Fourier transform infrared spectroscopy (FTIR) and the small angle X-ray scattering (SAXS). The morphological properties were assessed by scanning electron microscopy (SEM), and the water content of the collagen films after the DHT treatment was evaluated by thermogravimetry analysis (TGA).

## 2. Experiment

### 2.1. Materials

Collagen gel extracted from the hide of a 2-year-old steer was used. Corium split from steer hide was finely grounded into a paste, and natural cellulose fiber, about 77 μm in diameter, was added in it. Collagen was swollen in aq. HCl after grinding and homogenizing the paste. The paste was further homogenized into the collagen gel (pH ≈ 2) composed of the type I collagen (4.7 wt%), cellulose fiber (1.1 wt%), and HCl (0.2 wt%). 

The pure collagen gel was also prepared in the same way without adding cellulose fiber. The pure gelatin film from bovine bone was supplied by NITTA GELATIN INC. (Osaka, Japan), the thickness of which was about 1.1 mm. Aqueous ammonia solution (28%, 14.9 M) and urea (99.0 wt%) were purchased from NACALAI TESQUE, INC. (Kyoto, Japan).

### 2.2. Preparation of Collagen Film

About 6.5 g of collagen gel was put on the glass plate with a 0.5 mm thick spacer. Then, the collagen gel was squeezed and spread to a direction (MD) by using another glass plate. The surfaces of these glass plates are covered with a polyester film to prevent the adhesion of the gel to the plates. The gel block was finally completely spread into a homogeneous gel film of 50 mm × 150 mm × 0.5 mm. The collagen gel film on the glass frame was immersed in an excess amount of ammonia solution (0.37 M, pH ≈ 12) at room temperature for 10 min to neutralize and solidify. During the whole immersing process, the pH value of the ammonia solution was almost unchanged at around 12. Then, the film was taken out and washed exhaustively with deionized (DI) water for desalination and pH adjustment until pH = 7. The washed film was air-dried at room temperature for 24 h. The thickness of the dry collagen film was about 0.06 mm. The pure collagen film was prepared according to the same procedure described above. Pure collagen film and pure gelatin film were then used as references for Fourier transform infrared spectroscopy measurements.

### 2.3. Dehydrothermal Treatment

The dehydrothermal (DHT) treatment was performed as follows. Dry collagen film was kept in a vacuum oven (Yamato ADP 200, Yamato Scientific Co., Ltd, Tokyo, Japan) under a vacuum 0.5 kPa for 24 h for removing moisture. Then, the film was heated at various temperatures (85 °C, 105 °C, 125 °C and 145 °C) for 1 day and at 105 °C for the periods from 1 to 7 days at 1-day intervals. After the DHT treatment, films were cooled slowly to room temperature under vacuum.

The films were stored in a glass desiccator with a controlled atmosphere of 20 ± 2 °C and 50% ± 2% RH at least 48 h before analysis.

### 2.4. Characteristics

#### 2.4.1. Mechanical Properties

Mechanical properties of the collagen films in a wet state were determined by using a tensile tester (STA-1150, ORIENTEC, Tokyo, Japan). Film samples of 5 mm × 50 mm along the MD and TD (transverse direction) directions were prepared. The swollen films were prepared by immersing the film in DI water at room temperature for 24 h. After immersion, the film was taken out and the water on the surface was wiped with a filter paper before testing. The swollen film with a 20 mm clamping distance was stretched at a tensile rate of 30 mm/min at room temperature. The thickness of the swollen film was measured by using a digital micrometer after wiping water with a filter paper. After obtaining the stress-strain curve of a sample given directly from the tester, the tensile strength and the elongation to break were determined directly from the break point of the curve and the cross-section. The tensile modulus was the slope of the beginning part of curve near the alignment given by the software that accompanied the tensile tester. The rupture work was the area of curve from the starting point and the breaking point and was calculated by using Origin 8.0 software (OriginLab, Northampton, MA, USA). The measurements were repeated six times for each sample from more than four films.

#### 2.4.2. Water Content

Water content of the collagen films was determined by obtaining the thermogravimetric analysis (TGA) curves (TGA, Discovery TGA, Tokyo, Japan) as soon as the DHT treatment was finished. The samples around 20 mg were heated from room temperature to 400 °C with a heating rate of 10 °C/min under a nitrogen atmosphere. Differential thermogravimetric (DTG) analysis curves were obtained with the TGA data by numerically differentiating the later with respect to temperature. According to the TGA curves and DTG curves (Figure S1), the weight loss began to keep at about 182 °C. Therefore, water content was calculated based on the weight loss from room temperature to 182 °C.

#### 2.4.3. Morphological Analysis

The surface morphology of the collagen films was observed by using a scanning electron microscope (SEM, KEYENCE, Osaka, Japan, VE-7800) at 10 kV. The samples were coated with an ultra-thin layer of gold (IB-2, Eiko Engineering, Co. Ltd. Yamazaki, Japan) prior to the observation.

#### 2.4.4. Swelling Ratio

The swelling ratios of the collagen films were determined gravimetrically by immersing the film in DI water and in aq. urea (6 M) at 30 °C for 7 days. The immersion liquids were exchanged every two days to avoid bacterial breeding. The wet weight of the films was measured after wiping the immersion liquid on the surface with a filter paper. The films were air-dried at the same condition for 24 h after washing in water for 24 h to get the weight in dry state. The measurements were repeated five times for each sample from more than four films. The swelling ratio (*SR*) is defined as

(1) $$SR=\frac{M_{wet}-M_{dry}}{M_{dry}}$$

where *M_dry_* and *M_wet_* are the weights of the sample in a dry and wet state, respectively.

#### 2.4.5. Fourier Transform Infrared Spectra (FTIR)

The change of chemical structure of the collagen film was evaluated by Fourier transform infrared spectroscopy (FTIR, Spectrum GX, Perkin Elmer, Waltham, MA, USA) equipped with an ATR device with a Ge crystal plate. The spectra were recorded from 4000 to 750 cm^−1^ with an average of 16 scans and a resolution of 4 cm^−1^. The equilibrium moisture content of the samples before the FTIR testing was around 12%.

#### 2.4.6. Small Angle X-Ray Scattering (SAXS)

The higher order structure of the collagen was analyzed by small angle X-ray scattering (SAXS). SAXS patterns and spectra were obtained by using an X-ray generator MicroMax-007HF (Cu target), from Rigaku (Tokyo, Japan), operated at 40 kV and 30 mA, and a PILATUS 100K (Dectris, Baden, Switzerland) as a detector. The wavelength of the X-ray was 0.1542 nm and the camera length was 852 mm.

## 3. Results and Discussion

### 3.1. Mechanical Properties

The mechanical properties of the sausage casing have a great influence, not only on the efficiency of the meat-stuffing process in the food industry, but also on the mouthfeel of the sausage. For example, a high strength is necessary for the stable meat stuffing, and a high tensile modulus gives the sausage a “cracking bite”. However, the strength should be low enough that the casing disappears immediately after chewing in the mouth.

In this study, the effects of the DHT conditions, including the time (1~7 days) and temperature (85~145 °C), on the mechanical properties of the collagen films in a wet state were investigated systematically.

#### 3.1.1. Effect of DHT Temperature

**Figure 1:**
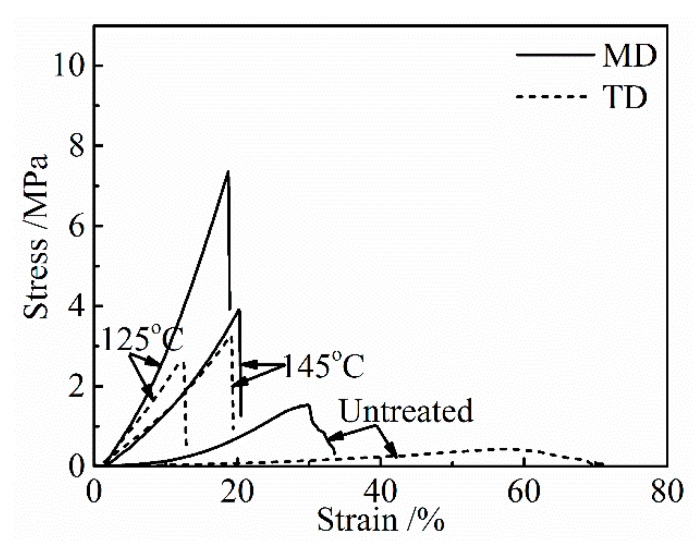
Stress-strain curves of the collagen films after applying the Dehydrothermal (DHT) treatment at various DHT temperatures for 1 day.

The stress-strain curves of the collagen films after applying a DHT treatment at various temperatures for 1 day are shown in Figure 1. The untreated collagen film shows a poor mechanical property, and a significant directional anisotropy was observed. Tensile strength and modulus are much higher, and the elongation to break is lower in MD, indicating a preferred molecular orientation in MD. The preferred orientation of collagen fibrils to MD in the collagen film, which was artificially prepared, has been confirmed by using SAXS [9]. With an increasing DHT temperature, strength and modulus increased and elongation to break decreased, and after showing the maxima in strength and modulus, they tended to decrease after the application of the DHT treatment at higher temperatures.

**Figure 2:**
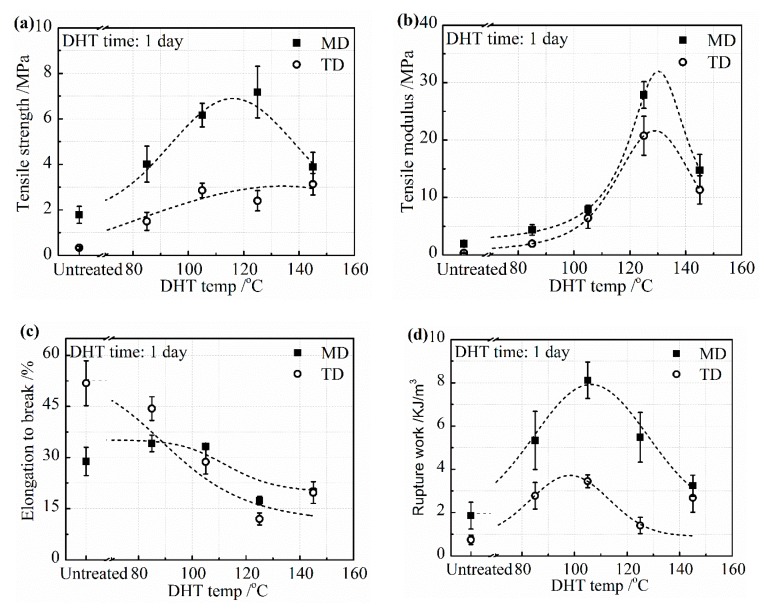
Mechanical properties: (**a**) tensile strength; (**b**) tensile modulus; (**c**) elongation to break; and (**d**) the rupture work of the collagen films after applying DHT treatment at various DHT temperatures for 1 day.

The mechanical properties, including tensile strength, tensile modulus, elongation to break, and the rupture work of the collagen films in a wet state are shown in Figure 2 in both MD and TD. The tensile strength was much higher in MD than in TD due to the orientation of collagen molecules caused by the method of preparation of the collagen film. The tensile strength and tensile modulus in MD and TD directions showed an increasing tendency up to the DHT temperature of 125 °C, as shown in Figure 2a,b, indicating that the DHT treatment resulted in the reinforcement of the collagen film by the formation of crosslinking. The tensile strength and tensile modulus increased to more than three times higher than that of the untreated film. However, it fell down, noticeably, when the DHT treatment was applied at 145 °C. It is considered to be attributed to the denaturation of collagen. The elongation to break decreased monotonically with an increasing DHT temperature, as seen in Figure 2c. Because of these changes, the rupture work reached the maximum value at the DHT temperature of 105 °C and fell down at higher DHT temperatures, as seen in Figure 2d.

#### 3.1.2. Effect of DHT Time

**Figure 3:**
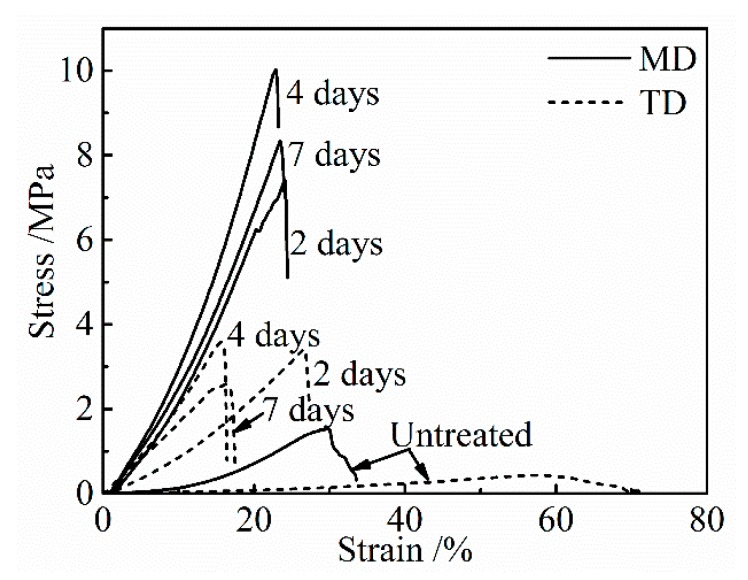
Stress-strain curves of the collagen films after applying DHT treatment for various DHT times at 105 °C.

Figure 3 shows the stress-strain curves of the untreated collagen film and those after the application of a DHT treatment at 105 °C for various periods of time. With an increasing DHT time, strength and modulus increased and elongation to break decreased, and after showing the maxima in strength and modulus they tended to decrease with a longer DHT time.

**Figure 4:**
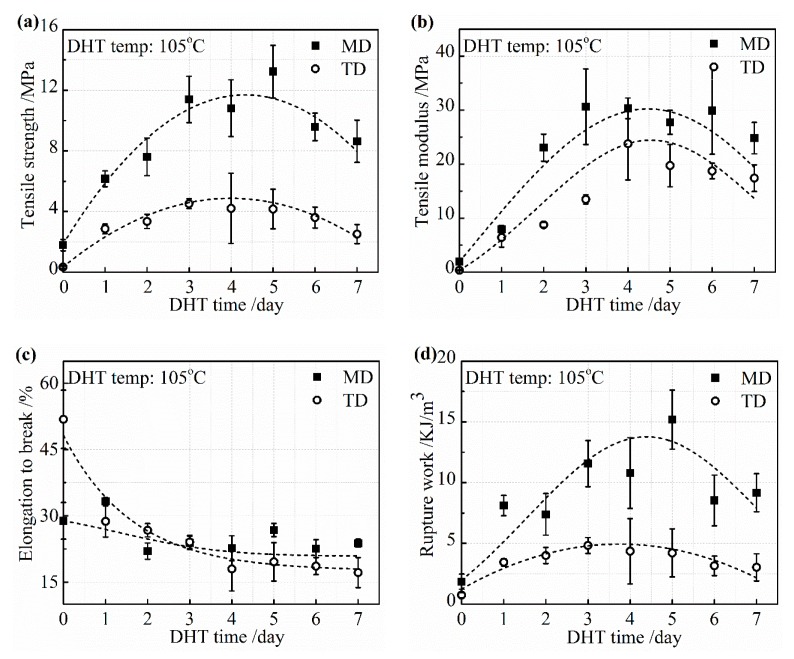
Mechanical properties: (**a**) tensile strength; (**b**) tensile modulus; (**c**) elongation to break; and (**d**) rupture work of the collagen films after applying DHT treatment at 105 °C for various DHT times.

Change in the mechanical properties of the collagen films with a DHT treatment at 105 °C are described in detail in Figure 4a–d. The tensile strength and tensile modulus increased rapidly with an increasing DHT time in both MD and TD in 3 days. Then, the increasing speed showed the plateau phase. The tensile strength began to reduce when the DHT time was longer than 5 days. A similar change tendency in the tensile modulus was obtained. The elongation to break monotonically decreased with an increasing DHT time and reached a constant value. The reduction of the elongation to break by the application of DHT may be due to not only the elimination of water, which acts as the plasticizer, but also because of the crosslinking between collagen molecules, which restricts the intermolecular mobility. The variation of tensile strength, tensile modulus, and elongation to break, with an increasing DHT time, resulted in the rupture work going up first and down with DHT time.

DHT treatment promoted the formation of crosslinking, which connects the collagen peptide chains and the tensile strength, and modulus improved noticeably with an increasing DHT temperature and time up to certain levels. When these parameters exceed the optimal value, the mechanical property tended to be poorer because of the denaturation.

### 3.2. Crosslinking

As analyzed above, it is indicated that DHT treatment introduces the crosslinking to improve the mechanical property of collagen films. Therefore, it is meaningful and crucial to clarify the crosslinking type, which is of great importance to the application of collagen films after DHT treatment.

Urea, as a protein denaturant, is a tool widely used to analyze protein stability. Through molecular dynamics simulations, Julian et al. had found that urea destroys the hydrogen bonding system that originally exists, which stabilizes the protein conformation [20]. Simultaneously, urea interacts with polar residues of the peptide backbone via hydrogen bonds, resulting in the loss of the natural structure of the protein [20,21]. The noncovalent hydrogen bonding in the protein is destroyed in aq. urea at a high concentration and the protein swells significantly [22]. On the other hand, the covalent crosslinking in the protein defends against the attack from urea and keeps the higher-order structure of the protein. In DI water, both types of the crosslinking can exist in the swollen collagen. Therefore, the fractions of the covalent and noncovalent crosslinking in the collagen can be determined by comparing the swelling ratios of collagen film in DI water and in aq. urea.

#### 3.2.1. Effect of DHT Temperature

**Figure 5:**
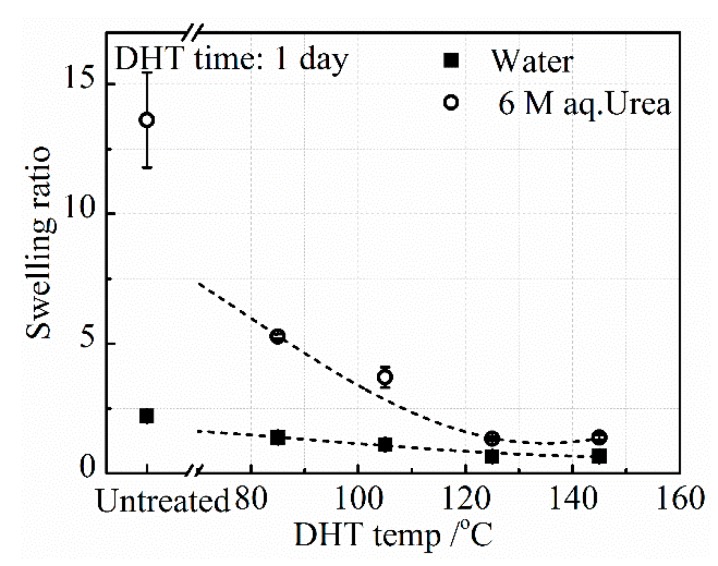
Change in *SR_urea_* and *SR_water_* with an increasing DHT temperature for 1 day.

The swelling ratios of the collagen films in DI water, *SR_wate_*_r_, and aq. urea, *SR_urea_*, after applying a DHT treatment at various temperatures for 1 day, are shown in Figure 5. It has been known that the swelling ratio is inversely proportional to the crosslinking density [23]. *SR_water_* of the untreated collagen film is as low as 2.5, while *SR_urea_* is about 13.5. Since urea breaks the hydrogen bonding in the collagen, the crosslinking that remains in the collagen is covalent bonding. This result indicates that both covalent and hydrogen bonded crosslinks exist in the collagen. 

The DHT treatment made a slight further reduction of *SR_water_*, indicating that additional crosslinking was introduced upon applying the DHT treatment and *SR_water_* slightly and monotonously decreased with DHT temperature. On the other hand, *SR_urea_* drastically decreased and approached the value of *SR_water_* with an increasing DHT temperature. These results suggest that the crosslinking introduced upon applying the DHT treatment is mainly covalent bonding between peptide chains, although some hydrogen bonded crosslinking is also introduced. According to Yannas et al., these chemical crosslinking bonds may be formed by the condensation reactions of either esterification or amidation [15]. This may be understandable, since DHT treatment heats the collagen film at elevated temperatures under vacuum, meaning that a condensation reaction is ready to occur. The amino acid residues involved in the condensation reactions are in glutamic acid, aspartic acid, lysine acid, arginine acid, serine acid, and threonine acid. One type I collagen molecule in this work constitutes about 24% (α1:744, α2:321) of the related residues of the total residues (sum: 4290) [24,25].

#### 3.2.2. Effect of DHT Time

**Figure 6:**
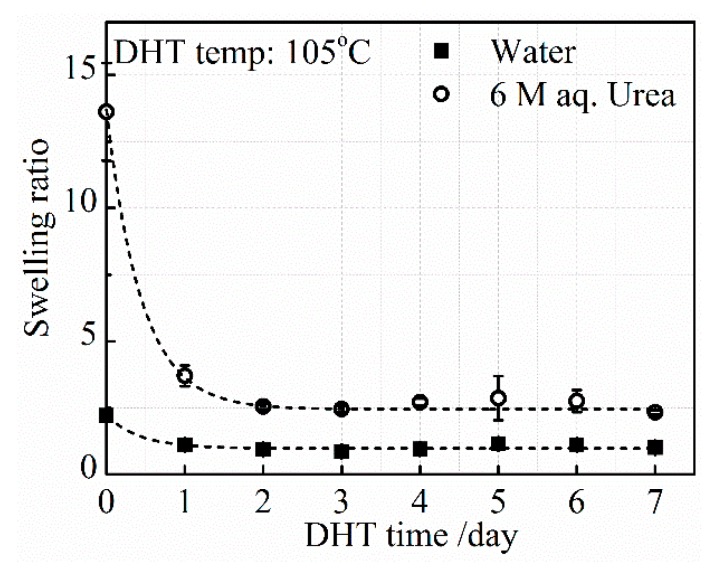
Change in *SR_urea_* and *SR_water_* with an increasing DHT time at 105 °C.

*SR_urea_* and *SR_water_*, after applying a DHT treatment at 105 °C for various DHT times, are shown in Figure 6. As already described in Figure 5, the level of *SR_urea_* in the untreated film is much higher than the level of *SR_water_*. After the application of the DHT treatment at 105 °C for 1 day, both *SR_urea_* and *SR_water_* decreased significantly, although the difference between *SR_urea_* and *SR_water_* is still large. The decreasing tendency of both *SR_urea_* and *SR_water_* continued until 3 days of DHT treatment, and these stayed constant at a longer DHT time. The density of covalent crosslinking increased with DHT time at 105 °C in 3 days. However, a further application of DHT treatment did not change the ratio between hydrogen-bonded and covalent crosslinking densities at 105 °C.

### 3.3. Denaturation

Water contained in the collagen film, including free- and hydrogen-bonded water, was mostly removed upon heating under vacuum in the DHT treatment. The water that remains in the collagen film plays an important role in maintaining the stability of the collagen triple helix structure by forming hydrogen bonded crosslinking [26]. Ravikumar et al. elucidated that the loss of water bridges is responsible for the unwinding of the collagen molecules by using molecular dynamics simulations, which further affects the collagen self-assembly [27]. 

**Figure 7:**
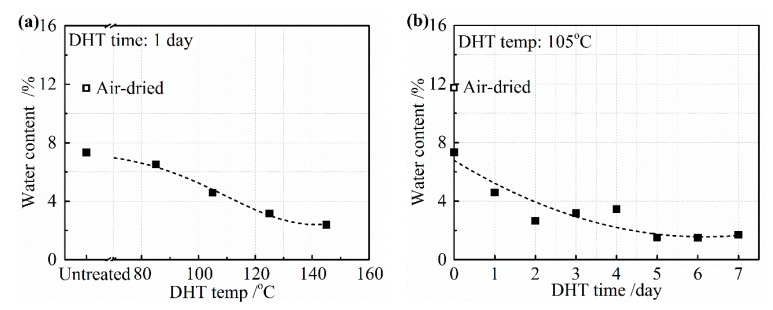
Water content in the collagen films after applying DHT treatment (**a**) at various DHT temperatures for 1 day and (**b**) at 105 °C for various DHT times.

Figure 7a,b depicts the water content from the collagen film after the application of a DHT treatment for (a) 1 day at various temperatures and (b) at 105 °C for various DHT times. The untreated film was only air-dried at room temperature and the film was further vacuum-dried before applying the DHT treatment. The air-dried film contained about 12 wt% of water and it reduced to about 7 wt% after vacuum-drying at room temperature. The water content gradually reduced with an increasing DHT temperature for 1 day, as shown in Figure 7a. About 2 wt% of water still remained, even after DHT treatment applied at 145 °C. Under the different periods of DHT time at 105 °C, about 5 wt% of water remained after 1 day. It reduced gradually to about 2 wt% for a longer DHT time in Figure 7b.

The removal of water results not only in the formation of crosslinking, but also in the damage of the collagen structure. So, the optimum DHT condition, in which an appropriate amount of crosslinking is introduced without causing severe denaturation, has to be determined. In this research, the extent of denaturation was monitored by the changes in the chemical structure and in the higher-order structure of collagen via FTIR-ATR and SAXS.

**Figure 8:**
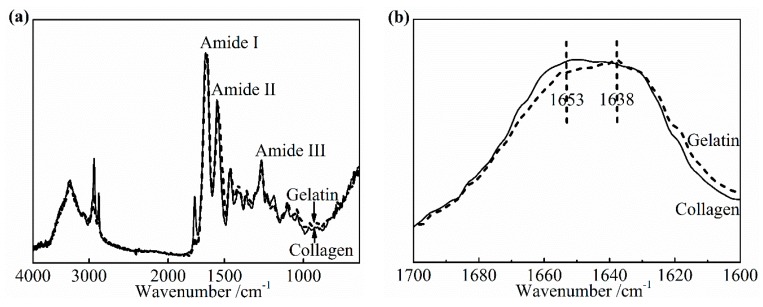
Fourier transform infrared spectroscopy (FTIR-ATR) spectra (**a**) and amide I band (**b**) of pure collagen film and gelatin film.

The significant advance has been achieved in the study of the characteristic band of collagen IR spectra, including the amide A band (~3300 cm^−1^), the amide I band (~1650 cm^−1^), the amide II band (~1550 cm^−1^), and the amide III band (~1240 cm^−1^) [28,29]. The IR amide I band is assigned to the stretching vibration of the peptide carbonyl group and has been widely used for the conformational analysis of the collagen owing to its sensitivity to the heterogeneity of carbonyl stretching modes in the backbone [30]. To determine the specific change in the amide I band as the denaturation occurs, an IR spectra of pure collagen film and gelatin film were obtained, as shown in Figure 8a,b. Gelatin is a hydrolyzed form of collagen losing the native triple helix structure partially or completely. Its chemical composition is closely similar to that of the parent collagen. The variation in the amide I band was clearly presented as the collagen turned into gelatin in Figure 8b. The center of the amide I band of gelatin spectra shifts to the low wavenumber. The relative intensity of the IR absorption of gelatin near 1653 cm^−1^ is much lower than that of collagen, which suggests that this part contained a component correlated to the native triple helix structure directly. In addition, a slightly higher absorption of gelatin, at about 1638 cm^−1^, may be assigned to non-triple helix components. Based on these changes from collagen to gelatin, the change of the collagen triple helix structure and the extent of denaturation upon applying the DHT treatment were analyzed.

#### 3.3.1. Effect of DHT Temperature

**Figure 9:**
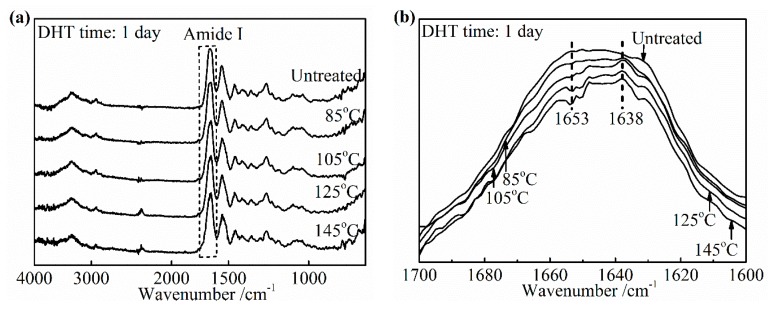
FTIR-ATR spectra (**a**) and amide I band (**b**) of the collagen films after applying DHT treatment at various DHT temperatures for 1 day.

Figure 9a,b shows the changes in the IR spectra and the amide I band of the DHT treated collagen films, respectively after applying DHT treatment for 1 day at various temperatures. The noteworthy features were observed directly among these peaks at 1653 cm^−1^ and 1638 cm^−1^ in Figure 9b. The DHT treated collagen films showed a significantly reduced intensity of the peak at 1653 cm^−1^, comparing with the spectra of the untreated collagen film. Moreover, the extent of reduction increased with an increasing DHT temperature. In contrast, the absorption at 1638 cm^−1^ became intensified apparently after DHT treatment. Because of these changes, the central of amide I band shifted to a lower wavenumber with an increasing DHT temperature. These characteristics were highly consistent with the phenomenon that the native collagen turned into denatured gelatin, which indicated that the native triple helix structure lost during DHT treatment partially. The degree of denaturation was low when the DHT temperature was 85 °C and it was much more serious with an increasing DHT temperature. In addition, the loss of the collagen triple helix may allow the peptide chains to form additional crosslinks. This would give rise to the increase in modulus, thereby decreasing elongation to break. It backed up the data in Figure 2b, which showed the tensile modulus increased by more than three times from 105 °C to125 °C in DHT treatment.

#### 3.3.2. Effect of DHT Time

**Figure 10:**
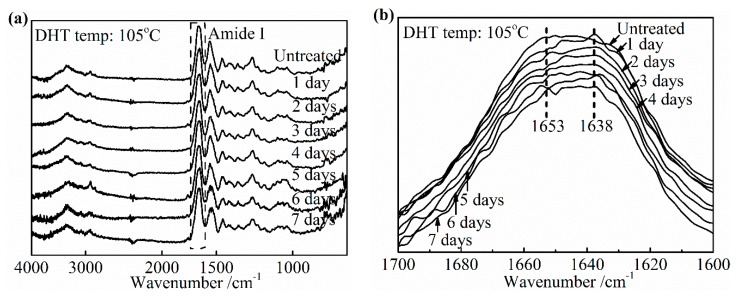
FTIR-ATR spectra (**a**) and amide I band (**b**) of the collagen films after applying a DHT treatment at 105 °C for various DHT times.

The similar variation tendency of IR spectra as a function of DHT treatment time is observed in Figure 10. It was found that the intensity at about 1653 cm^−1^ related to the native triple helix structure diminished compared with the untreated film in Figure 10b. Currently, the absorption of nearby 1638 cm^−1^ became stronger. When DHT time exceeded 5 days, the central of amide I band of treated collagen films shifted to lower wavenumber obviously, which showed the DHT treatment brought the serious denaturation. In Figure 6, it had found that the density of crosslinking kept unchanged after 3 days at 105 °C. The combination of these two points makes it clear that the tensile strength shown the plateau phase from 3 to 5 days and began to decrease after 5 days. However, the increasing trend of the denaturation with DHT time is not clear unlike the effect of DHT temperature.

#### 3.3.3. Change of the Higher Order Structure and the Surface Morphology

**Figure 11:**
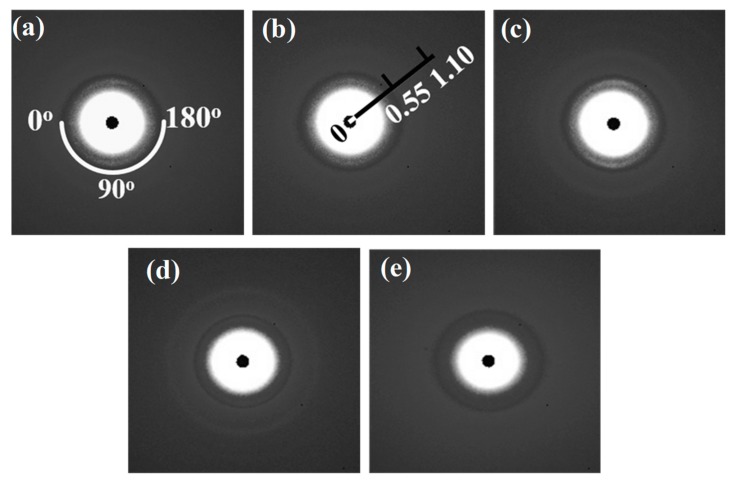
2D SAXS patterns. The untreated film (**a**); collagen film treated at 105 °C for 3 days (**b**); 105 °C for 7 days (**c**); 125 °C for 1 day (**d**) and 145 °C for 1 day (**e**).

**Figure 12:**
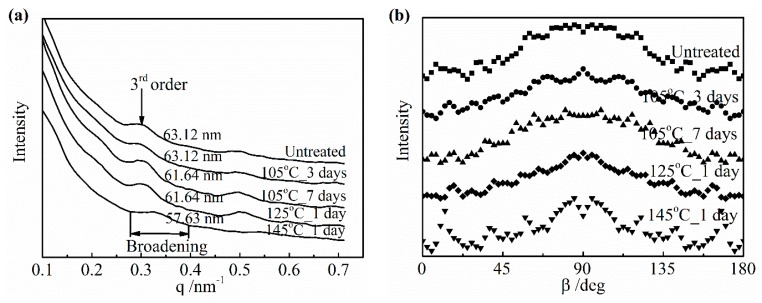
Meridional (**a**) and azimuthal plots (**b**) of small angle X-ray Scattering (SAXS) spectra.

**Figure 13:**
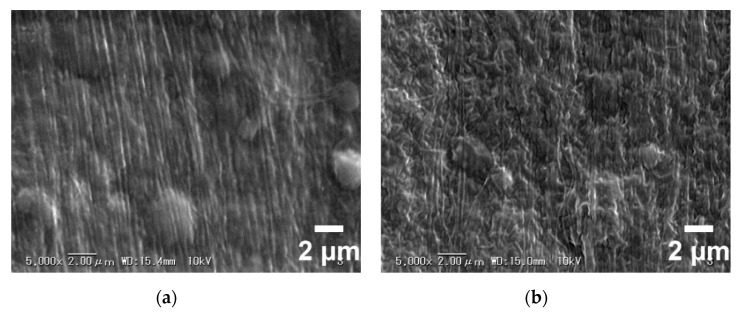
Scanning electron microscopy (SEM) images of (**a**) the untreated film and (**b**) the collagen film treated at 145 °C for 1 day.

The 2D SAXS patterns of the collagen films with representative DHT treatments are displayed in Figure 11. All of the patterns show a stronger diffraction at the meridional direction, due to a shear flow applied at the preparation of the film. For visualized and precise structural analyses, meridional and azimuthal plots of SAXS spectra were conducted in Figure 12a,b, respectively. From Figure 12a, the meridional plots were the scattering intensity relative to the scattering vector *q* at 90° azimuth along MD (*q* = (4π/λ) sin (θ/2), with λ and θ being the wavelength of an X-ray and the scattering angle, respectively). The position of the 3rd order shifted to a higher q after the application of a DHT treatment for a long period of time or at a high temperature. According to Bragg’s law, the periodicity distance D of collagen molecule packing could be evaluated specifically [6]. It can be seen that the length of periodicity shortened from 63.12 to 61.64 nm after the application of a DHT treatment at 125 °C for 1 day and at 105 °C for 7 days. Furthermore, it reduced to 57.63 nm as the DHT temperature increased to 145 °C. The SEM images show this phenomenon in a more intuitive way in Figure 13a,b. An obvious shrinkage of collagen fibrils was observed along MD in the DHT treated collagen film, whereas the collagen fibrils in the untreated film kept straight. In addition, the SAXS 3rd peak, after a DHT treatment at 145 °C, broadened compared with other profiles, perhaps attributed to the poor regularity of periodicity. These data indicate that the DHT treatment could lead to the damage of collagen molecular self-assembly by the breakage of original interactions between collagen molecules upon the application of a DHT treatment at a high temperature or for a long period time.

From the azimuthal plots of the 3rd order diffraction in Figure 12b, the orientation of collagen fibrils did not show a significant change along MD. Nevertheless, the overall distribution of the intensity of the collagen film treated at 145 °C was more discrete due to the discrete electron density of the repeated unit. This further explained that the DHT treatment could impact the arrangement of collagen molecules. 

## 4. Conclusions

The mechanical properties of the sausage casing made of collagen films are of great importance to the mouthfeel of sausage and the processability during meat stuffing. This research adopted a DHT treatment with different conditions to enhance the mechanical properties. The formation of a covalent crosslinking bond after analyzing the swelling ratios of collagen film in DI water and in 6 M aq. Urea was observed. Furthermore, the crosslinking density increased with an increasing DHT temperature, and time contributed to the continuous improvement of tensile strength. However, the loss of a native triple helix structure and the damage of the self-assembly of collagen molecules also occurred from the data of IR and SAXS, which decreased the mechanical properties. The collagen films show favorable mechanical properties with DHT treatment at 125 °C for 1 day or 105 °C for 3 days. A continuously increasing DHT temperature or time led to serious denaturation and the decrease of mechanical properties with a lower tensile strength, a higher tensile modulus, and a shorter elongation at a DHT temperature of 145 °C for 1 day or for a longer DHT time over 5 days at 105 °C.

## Supplementary Materials

The following are available online at https://www.mdpi.com/1996-1944/13/2/377/s1, Figure S1: TGA curves and DTG curves of collagen films with DHT treatment, (a) at various DHT temperatures for 1 day; (b) for various DHT time at 105 °C.
