# Supplementary material for: Effect of the Application of a Dehydrothermal Treatment on the Structure and the Mechanical Properties of Collagen Film
Source: Materials (Basel). 2020 Jan 14;13(2):377. doi: 10.3390/ma13020377 (PMC7013574; doi:10.3390/ma13020377)
Supplement: Supplementary file 1 [file materials-13-00377-s001.pdf]

materials

Supplementary Materials

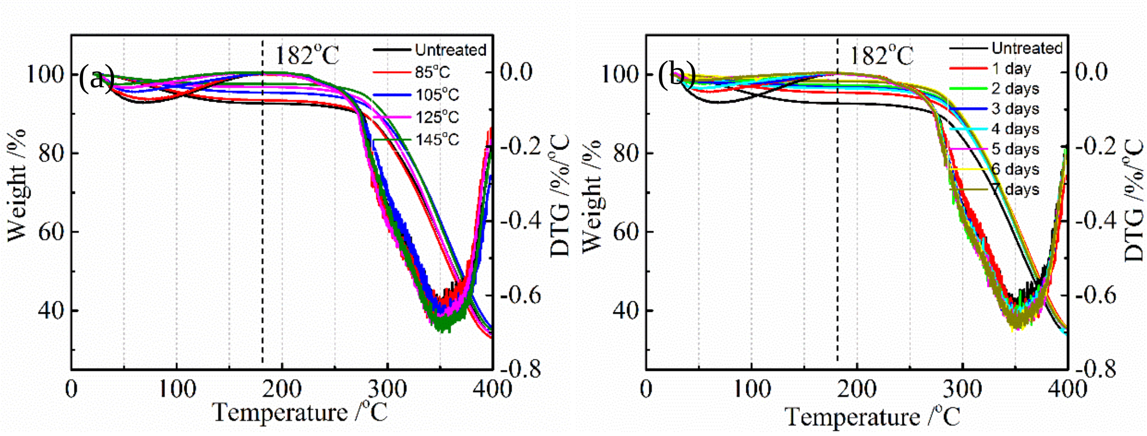

Figure S1. TGA curves and DTG curves of collagen films with DHT treatment, (**a**) at various DHT temperatures for 1 day; (**b**) for various DHT time at 105 °C.
